# Hydroxycarboxylic Acid Receptor 2, a Pleiotropically Linked Receptor for the Multiple Sclerosis Drug, Monomethyl Fumarate. Possible Implications for the Inflammatory Response

**DOI:** 10.3389/fimmu.2021.655212

**Published:** 2021-05-18

**Authors:** Benedetta Parodi, Alessia Sanna, Alessia Cedola, Antonio Uccelli, Nicole Kerlero de Rosbo

**Affiliations:** ^1^ Neuroimmunology Laboratory, DINOGMI, University of Genoa, Genoa, Italy; ^2^ CNR-Institute of Nanotechnology, Rome, Italy; ^3^ IRCCS Ospedale Policlinico San Martino, Genoa, Italy

**Keywords:** multiple sclerosis, dimethyl fumarate, hydroxycarboxylic acid receptor 2, experimental autoimmune encephalomyelitis, dendritic cells, intestinal epithelial cells

## Abstract

Monomethyl fumarate (MMF), metabolite of dimethyl fumarate (DMF), an immunosuppressive drug approved for the treatment of multiple sclerosis (MS), is a potent agonist for hydroxycarboxylic acid receptor 2 (HCAR2), eliciting signals that dampen cell activation or lead to inflammation such as the skin flushing reaction that is one of the main side effects of the treatment, together with gastrointestinal inflammation. Our aim is to further understand the molecular basis underlying these differential effects of the drug. We have used wild-type and HCAR2 knock-out mice to investigate, *in vitro* and *ex vivo* under steady-state and pathological conditions, the HCAR2-mediated signaling pathways activated by MMF in dendritic cells (DC), which promote differentiation of T cells, and in intestinal epithelial cells (IEC) where activation of a pro-inflammatory pathway, such as the cyclooxygenase-2 pathway involved in skin flushing, could underlie gastrointestinal side effects of the drug. To understand how DMF treatment might impact on gut inflammation induced by experimental autoimmune encephalomyelitis (EAE), the animal model for MS, we have used 3D X-ray phase contrast tomography and flow cytometry to monitor possible intestinal alterations at morphological and immunological levels, respectively. We show that HCAR2 is a pleiotropically linked receptor for MMF, mediating activation of different pathways leading to different outcomes in different cell types, depending on experimental *in-vitro* and *in-vivo* conditions. In the small intestine of EAE-affected mice, DMF treatment affected migration of tolerogenic DC from lamina propria to mesenteric lymph nodes, and/or reverted their profile to pro-inflammatory, probably as a result of reduced expression of aldehyde dehydrogenase and transforming growth factor beta as well as the inflammatory environment. Nevertheless, DMF treatment did not amplify the morphological alterations induced by EAE. On the basis of our further understanding of MMF signaling through HCAR2, we suggest that the pleiotropic signaling of fumarate *via* HCAR2 should be addressed for its pharmaceutical relevance in devising new lead compounds with reduced inflammatory side effects.

## Introduction

Dimethyl fumarate (DMF) is an immunomodulatory drug originally used for the treatment of psoriasis, a type-1 cytokine-mediated chronic autoimmune skin disease aided by the infiltration of Th1/Th17 cells into the skin (1). It is also a first-line oral treatment for relapsing-remitting multiple sclerosis (MS), a chronic inflammatory autoimmune disease of the central nervous system characterized by demyelination and axonal damage and loss. The mechanism of action of DMF and monomethyl fumarate (MMF), its bioactive metabolite, is incompletely defined. The anti-inflammatory effects of DMF have been attributed to the possible activation of the nuclear factor (erythroid-derived 2)-like 2 anti-oxidant pathway (2–4), to the inhibition of pro-inflammatory pathways, such as nuclear factor kB (NF-kB) and ERK1/2, in dendritic cells (DC) (5, 6), and, more recently, to signaling *via* the hydroxycarboxylic acid receptor 2 (HCAR2) (7, 8), of which MMF is a potent agonist (9). HCAR2 is a Gi-protein-coupled receptor expressed on several cell types, including immune cells such as mature neutrophils, DC, and macrophages, but not B or T cells (9). Treatment with DMF ameliorates the clinical course of experimental autoimmune encephalomyelitis (EAE), the model for MS, in wild-type, but not HCAR2-deficient, mice by reducing the infiltration of neutrophils involved in disease progression (7). We have shown that MMF, which crosses the blood-brain barrier, might also act in EAE by modulating the activated phenotype of microglia through a new HCAR2-dependent pathway, whereby activation of HCAR2 by MMF prompts the activation of the AMP-activated protein kinase (AMPK)/Sirtuin1 (Sirt1) axis (HCAR2/AMPK-Sirt1 pathway) leading to the inhibition of the NF-kB pathway (8). Activation of HCAR2 can also lead to pro-inflammatory effects depending on the context and ligand. Indeed, stimulation of HCAR2 by MMF, or another HCAR2 agonist, nicotinic acid (NA), induces the cyclooxygenase-2 (COX-2)-mediated synthesis of prostaglandins (HCAR2/COX-2 pathway) in keratinocytes and Langerhans cells (10), which is responsible for skin flushing, one of the most common side effects associated with DMF treatment. *In-vitro* studies with immortalized human embryonic kidney cells have also shown that activation of HCAR2 by NA can trigger the activation of another pro-inflammatory pathway, the ERK MAPK pathway (HCAR2/ERK1/2 pathway) (11).

In this study, we investigated which signaling pathways activated by MMF binding to HCAR2 can underlie the contrasting anti- and pro-inflammatory effects of the drug, under different *in-vitro* and *in-vivo* conditions and in different cell types relevant to DMF effect in MS. In particular, we focused on DC, in view of their role in driving autoimmunity by promoting the differentiation of effector T cells (12), and on intestinal epithelial cells (IEC). Indeed, aside from skin flushing, the other most common side effect of DMF treatment is gastrointestinal manifestations in the first few months of treatment (13, 14), and we speculated that these could be related to the activation of HCAR2-triggered pro-inflammatory pathways, such as HCAR2/COX-2 or HCAR2/ERK1/2 pathways, in IEC. We used EAE as a model to investigate the possible effect of DMF treatment on the gut, at morphological and immunological levels, also considering the importance of the now recognized occurrence of intestinal inflammation in MS (15).

## Methods

### Mice

Wild-type (WT) C57BL/6J mice, originally purchased from Charles River, were maintained in our own colony. HCAR2 knock-out (HCAR2-KO) mice were developed by Jackson Laboratory on the C57BL/6J background and were the kind gift of Biogen; the Hcar2 gene in these mice lacks a 507 bp region from bp 167 to bp 673, resulting in an in-frame deletion and the abrogation of HCAR2 at mRNA (data not shown) and protein (**Supplementary Figure 1**) levels. HCAR2-KO mice were viable, healthy, fertile, and indistinguishable from WT mice. All animals were housed in pathogen-free conditions with food and water ad libitum and treated according to the Italian and European guidelines (Decreto Legislativo 4 marzo 2014, n. 26, legislative transposition of Directive 2010/63/EU of the European Parliament and of the Council of 22 September 2010 on the protection of animals used for scientific purposes). The research protocol for collecting cells from naïve mice and for the EAE experiments (see below) was approved by the Animal Ethics Committee of IRCCS Ospedale Policlinico San Martino and by the Italian Ministry of Health (Approval Number: 398; authorization n. 679/2016-PR).

### DC Cultures and Treatments

Bone marrow-derived DC (BM-DC) were isolated from femurs and tibias of 8-10-week-old C57BL/6J WT mice as previously described (16). Briefly, at day 0, BM cells were flushed out and the cell suspension was filtered through a 100-µm cell strainer (Becton–Dickinson) and seeded (1 x 10^6^ in 6-well plates) in RPMI (containing 10% fetal bovine serum (FBS), 1% non-essential amino acids, 1% Na pyruvate, 1% penicillin/streptomycin) in the presence of granulocyte macrophage colony-stimulating factor (20 ng/ml, Miltenyi). Half of the medium was replaced on alternate days. At day 7, the purity of CD11c+ cells assessed by flow cytometry with PE-conjugated anti-CD11c antibody (1:100, Biolegend cod. 117308) was at least 70%. To isolate splenic DC (sDC), spleens were excised from 8-10-week-old C57BL/6J WT and HCAR2-KO mice and mechanically minced through a cell strainer (100-μm pore size, BD Falcon) and the cell suspension was subjected to MACS Column technology using CD11c microbeads (Miltenyi Biotec) according to the manufacturer’s instructions. CD11c+ cell purification was conducted over MS columns on a MiniMACS separator (Miltenyi Biotec) and the purity assessed by flow cytometry as above was at least 85%. Cells were seeded (2 x 10^5^ in 96-well plates) in RPMI supplemented with 2% mouse serum, 1% non-essential amino acids, 1% Na pyruvate, 1% penicillin/streptomycin.

Both BM-DC and sDC were activated with 100 ng/ml lipopolysaccharide (LPS) and simultaneously treated with MMF (10 or 25 μM) for the times indicated in the text and/or figure legends. The selected MMF concentrations are commensurate with the median MMF daily plasma concentration in patients with MS treated with DMF (approximately 13 μM) (17). Cells were detached using ice-cold PBS containing 2 mM ethylenedinitrilo-tetraacetic acid (EDTA, Sigma), pelleted for Western blotting or resuspended in Qiazol for PCR, and stored at -80°C.

### IEC Culture and Treatments

IEC were cultured as isolated crypts from the ileum of 8-10-week-old C57BL/6J WT and HCAR2-KO mice as described (18), with minor modifications. Briefly, the ileum was cut in three sections that were opened lengthwise, washed with PBS containing 100 U/ml penicillin, 100 mg/ml streptomycin and 25 ng/ml amphotericin B (Sigma), and cut into 1-2 mm pieces. After several washing steps, the fragments were incubated with EDTA (2 mM) on a shaker for 15 min at 37°C, to enable the detachment of the crypts from the mucosa. This step was repeated 3-4 times without shaking using resuspension with a pipette, and the suspension was filtered on 100-µm cell strainers. At each step, the suspension step was checked by optical microscope to select crypts that were at least 20-50 µm. These were resuspended in medium containing Matrigel (Corning) (ratio medium:Matrigel 1:1) and seeded in 24-well plates at the concentration of 300 crypts per well for the *in-vitro* experiments. They are thereafter referred to as IEC. IEC culture medium was composed of: DMEM/F12 (Thermo Fisher), 2% FBS (Thermo Fisher Scientific), 0.2% D-glucose (Sigma), 2 mM L-glutamine (Thermo Fisher Scientific), 20 mM HEPES (Sigma), 100 U/ml penicillin, 100 mg/ml streptomycin, 5 ug/ml of insulin (Sigma), 5 ug/ml transferrin (Sigma), 50 ng/ml mEGF (mouse epidermal growth factor, Sigma), 100 ng/ml mNoggin (Peprotech), 500 ng/ml R-spondin-1 (Peprotech). IEC were cultured at 37°C in a 5% CO_2_ atmosphere for 2 days. *In-vitro*-cultured IEC were stimulated or not with interferon-γ (IFNγ) and treated with MMF (25 μM) or with Butyrate (10 mM) for 6 hours, as indicated in the text and/or figure legends. For ex-vivo experiments, crypts were isolated as above, but immediately pelleted for Western blotting or resuspended in Qiazol for PCR, and stored at -80°C.

### RNA Isolation and Real-Time PCR

Total RNA was isolated using Qiazol reagent (Qiagen) according to the manufacturer’s instructions. cDNA was synthetized from 1 μg RNA using the QuantiTect Reverse Transcription Kit (Qiagen). Real-time PCR was performed using a LightCycler 480 (Roche) in duplicates with a final reaction volume of 20 μl containing 50 ng cDNA, 1 μl of each primer pair (20 μM), and 10 μl of LightCycler 480 FastStart Essential DNA Green Master (Roche). Amplification of glyceraldehyde 3-phosphate dehydrogenase (Gapdh) mRNA was used to normalize the expression data. The primer pairs for the indicated genes were synthetized (Tib Molbiol) according to the following sequences: Tnf, forward primer: 5′-TCTTCTCATTCCTGCTTGTGG-3′, reverse primer: 5′-GGTCTGGGCCATAGAACTGA-3′; Il1b, forward primer: 5′-AGTTGACGGACCCCAAAAG-3′, reverse primer: 5′-TTTGAAGCTGGATGCTCTCAT-3′; Il12, forward primer: 5′- CCAGGTGTCTTAGCCAGTCC-3’, reverse primer: 5′- GCAGTGCAGGAATAATGTTTCA-3’; Il23: forward primer: 5′- TCCCTACTAGGACTCAGCCAAC-3’, reverse primer: 5′- AGAACTCAGGCTGGGCATC-3’; Hcar2: forward primer: 5′- CTGCTCAGGCAGGATCATCT-3’, reverse primer: 5′- CCCTCTTGATCTTGGCATGT-3’; Aldh1a2: forward primer: 5’- CATGGTATCCTCCGCAATG-3’; reverse primer: 5’-GCGCATTTAAGGCATTGTAAC-3’; Tgfb1: forward primer: 5’-TGGAGCAACATGTGGAACTC-3’; reverse primer: 5’-GTCAGCAGCCGGTTACCA-3’; Cdh1: forward primer: 5′- ATCCTCGCCCTGCTGATT-3′, reverse primer: 5′- ACCACCGTTCTCCTCCGTA-3′; Ccl19: forward primer: 5′- TGTGGCCTGCCTCAGATTAT-3′, reverse primer: 5′- AGTCTTCCGCATCATTAGCAC-3′. Ccr9: forward primer: 5′-GATGCCCACAGAACTCACAA-3′, reverse primer: 5′- CTGTGGAAGCAGTGGAGTCA-3′. Gapdh: forward primer: 5′-AATCTCCACTTTGCCACTGC-3′, reverse primer: 5′-ATGGTGAAGGTCGGTGTGA-3′.

### Fluorimetric Determination of Intracellular Ca^2+^ Concentration

sDC cells (3 × 10^5^) were plated on 20-mm-diameter coverslips and activated, or not, with 100 ng/ml LPS in the presence or absence of 25 µM MMF for 1 h. Cells were then incubated with the fluorescent calcium indicator Fura-2-acetoxymethyl ester (Fura 2 AM) for 45 min at 37°C, and the intracellular Ca^2+^ concentration was measured as fluorescent intensity on an inverted microscope (Zeiss IM35, Zeiss), as described elsewhere (19).

### Western Blotting

Cells were lysed in RIPA buffer (Sigma) containing protease inhibitor cocktail (Roche) and phosphatase inhibitor cocktail (Sigma). Protein samples (15-30 μg) were electrophoresed on a 4–12% gradient polyacrylamide pre-cast gel (Thermo Fisher Scientific), using Bolt^®^ Mini Gel Tank (Thermo Fisher Scientific) and transferred to nitrocellulose membrane (BioRad) using XCell II™ Blot Module (Thermo Fisher Scientific). Membranes blocked with 5% bovine serum albumin (BSA, Sigma) in PBS containing 0.1% Tween-20 were incubated with primary antibodies overnight, washed and incubated with horseradish peroxidase-conjugated anti-rabbit IgG (1:4000, Merck-Millipore, AP307P) for 1 h, and visualized using ECL Plus (Thermo Fisher Scientific). Quantification of relative protein amounts was performed by densitometric analysis using ImageJ software (NIH), normalized to GAPDH or α-tubulin (α-Tub) as loading control protein. Primary rabbit antibodies used were: anti-HCAR2 (1:1000, Abcam, cod. ab198693), anti-phospho-5′ adenosine monophosphate-activated protein kinase (P-AMPK) Thr172 (1:1000, Cell Signaling, cod. 2535), anti-phospho-nuclear factor κB (NF-kB) p65 subunit (P-p65, 1:1000, Cell Signaling, cod. 3033), anti-tot-NF-kB p65 (tot-p65, 1:2000, Cell Signaling, cod. 4764), anti-cyclooxygenase-2 (COX-2) (1:1000, Cell Signaling, cod.12282), anti-phospho-ERK1/2 (P-ERK1/2) Thr202/Tyr182 (1:2000, Cell Signaling, cod. 4370), anti-ERK1/2 (1:2000, Cell Signaling, cod. 4695), anti-α-Tubulin (α-Tub) (1:10000, Millipore, cod. MABT205) and anti-GAPDH (1:5000, Cell Signaling, cod. 2118) antibodies.

### EAE Induction and DMF Treatment

WT and HCAR2-KO C57Bl/6J female mice, 6–8 weeks old, weighing 18.5 ± 1.5 g, were immunized as previously described (20) by subcutaneous injection (200 µl total) at two sites in the flank with an emulsion of 200 μg myelin oligodendrocyte glycoprotein peptide 35–55 (Espikem) in incomplete Freund adjuvant (Difco) containing 600 μg Mycobacterium tuberculosis (strain H37Ra; Difco). Mice were injected in the tail vein (100 µl total volume) with 400 ng pertussis toxin (Sigma-Aldrich) immediately and 48 h after immunization. The mice were scored daily for clinical manifestations of EAE on a scale of 0–5 (20). The DMF suspension (15 mg/ml) in 0.8% hydroxypropyl methylcellulose was prepared weekly and kept at 4°C under constant stirring. DMF (200 μl corresponding to a dose of 150 mg/kg body weight) was administered daily by oral gavage through a bulb-tipped curved gastric gavage needle by trained operators from the onset of clinical symptoms (score 1) until day 4. Control EAE animals were gavaged with vehicle alone. Mice were treated and daily assessed in random order. For sampling and at completion of the experiment, mice were euthanized by gradual-fill CO_2_ exposure.

### Immunocytochemistry and Immunohistochemistry

IEC seeded on 20-mm glass coverslips (150 crypts per coverslip) were incubated overnight at 37°C, 5% CO_2_. The next day, cells were fixed with 4% paraformaldehyde (PFA) for 10 min at room temperature and permeabilized with 0.25% Triton X-100 for 10 min. After blocking in 1% BSA for 30 min at room temperature, cells were stained for 1 h in humid chamber with primary rabbit anti-HCAR2 (1:50, Abcam, cod. ab198693) and mouse anti-Pan-Keratin (1:400, Cell Signaling, cod. 4545) antibodies in 1% BSA. Cells were then incubated with secondary Alexa 488-labeled anti-rabbit and Alexa 594-labeled anti-mouse antibodies (used at 1:2000, Thermo Fisher Scientific, cod: A-21200 and A21044) at room temperature for 30 min and the nuclei were identified with 4,6-diamidino-2-phenylindole (DAPI). Images were obtained by fluorescence microscopy on Axio Imager.M1 (Zeiss).

Isolated ilea were washed with PBS and fixed in 0.2% glutaraldehyde and 2% PFA. After 24 h, tissues were transferred in 20% sucrose overnight and embedded in optimal cutting temperature (OCT) compound. The OCT blocks were sliced using a cryostat (slice thickness: 8 µm). After blocking in 1% BSA/PBS for 30 min at room temperature, slices were stained for 1 h in humid chamber with primary rat anti-CD45 antibody (1:100, Biolegend cod. 103102) in 1% BSA/PBS. Slices were then incubated with secondary Alexa 594-labeled anti-rat antibody (1:2000, Thermo Fisher Scientific, cod. A-11007) at room temperature for 30 min and the nuclei were identified with DAPI. Images were obtained by confocal microscopy on TCS SP5 (LEICA Microsystems GmbH).

### X-Ray Phase Contrast Tomography (XPCT) Data Acquisition and Quantification

Mice were sacrificed using CO_2_ and the ilea were isolated from the whole gut, fixed in 4% PFA for 24 hours and stored in 70% ethanol until acquisition of XPCT images. The XPCT experiment was carried out at the ID17 beamline of the European Synchrotron Radiation Facility (Grenoble, France). A peak of energy of 25KeV was chosen for the pink beam, taking into account the density and thickness of the samples (21). The detection system used was composed of a YAG-based scintillator screen coupled with a visible-light optics and a PCO.Edge5.5 sCMOS detector (2560 × 2160 pixels) (22). The study was carried out using 2x optical magnification systems to determine a final pixel size of 3.2 µm and FOV:(7.6 × 6.4 mm^2^). The spatial resolution, defined as the minimum distance between two objects in order to resolve them, is approximately twice the pixel size, i.e. 7 µm. The sample was set at a distance of 1.5 m from the detector and the acquisition was performed in free-space propagation mode. XPCT was acquired with 4000 projections and an exposure time of 0.06 s for each projection covering a total angle range of 360 degrees. Data processing procedures, including pre-processing of the detected projections (normalization to flat field and dark field) and processing (phase retrieval based on Paganin algorithm (23) and tomographic reconstruction with filter back projection]), were performed with SYRMEP Tomo Project software (24) implementing ASTRA toolbox for efficient tomographic reconstruction on GPU. The image post processing was performed using free software (ImageJ). A virtual flattening of the ileum was carried out using ImageJ Radial reslice plugin that creates orthogonal reconstructions of an image stack by rotating a line ROI around its midpoint. Starting from the flattened ileum, villus length was measured on well laid-out villi only. To ensure the accuracy of the measurements and ascertain that the flattening operation had not altered the size of the villi, the length of a few villi were verified on the unflattened ileum and the measurements were compared with those of the same villi made on the flattened ileum. Villus length was measured from the top of the crypt to the tip of the villus (25). The width measurements (major and minor widths) were made at the base of the villus, above the top of the crypt, by rescaling the image of the ileum wall to reduce alteration from the virtual flattening operation. As the villus can be approximated to a conical ellipsoid (26), we measured the transversal area as a x b x π with a = major radius and b = minor radius. Measurements were performed on approximately 120 villi per mouse, with n = 3 mice per group.

### Isolation of Lamina Propria (LP) and Mesenteric Lymph Node (MLN) Cells

LP cells were isolated as described (27), with minor modifications. Briefly, intestinal tissue fragments were obtained as above, exposed to EDTA (2 mM) and incubated on a shaker at 37°C for 15 minutes to remove the epithelial cells. The resulting fragments were digested in RPMI-1640 containing 5% FBS, 1 mg/ml collagenase D (Roche) and 5 U/ml DNase I (Sigma) for 40 min at 37°C on an orbital shaker. The digested fragments were filtered on 100-µm cell strainers and the resulting cell suspension was pelleted and resuspended in PBS containing 1% FBS for fluorescence-activated cell sorting (FACS) analysis.

MLN were isolated from the mesentery and digested in RPMI-1640 containing 5% FBS, 1 mg/ml collagenase D and 5 U/ml DNase I for 40 min at 37°C on a shaker, as described (28). The MLN were mechanically dissociated and the cell suspension was filtered on 100-µm cell strainers, pelleted, and resuspended in PBS containing 1% FBS for FACS analysis.

### FACS Analysis

LP and MLN cells were assessed by FACS for specific surface markers using PE-conjugated anti-CD11c (1:100, Biolegend, cod. 117308), FITC-conjugated anti-CD103 (1:100, Biolegend, cod. 121419), FITC-conjugated anti-CCR7 (1:100, Serotec, cod. MCA2367F), APC-conjugated anti-CD3 (1:100, Miltenyi, cod. 130-117-793) Pacific Blue-conjugated anti-CD4 (1:100, Biolegend, cod. 100428), and FITC-conjugated anti-CCR9 (1:100, Miltenyi, cod. 130-115-602) antibodies. Cells (5 × 10^5^) were resuspended in 100 μl PBS containing 0.5% BSA and stained with the antibodies for 20 min at room temperature. Intracellular staining on LP and MLN cells was performed using Cytofix/Cytoperm, Fixation/Permeabilization Solution Kit (Becton–Dickinson), according to the manufacturer’s protocol. Briefly, cells were incubated with FITC-conjugated anti-CD4 and APC-conjugated anti-CD25 (1:100, Biologend, cod. 101709) antibodies, fixed/permeabilized for 15 min at 4°C, and processed for intracellular staining using PE-conjugated anti-FoxP3 antibody (1:100, Biolegend, cod. 126403) for 30 min at 4°C. Data were acquired on a FACS Canto II (Becton–Dickinson) and analyzed using DIVA 6.1 software.

### Statistical Analysis

Results are presented as mean ± standard error of the mean (SEM). The difference in means between two groups was assessed by two-tailed Student’s t-test, while the difference in means between three or more groups was assessed by one-way ANOVA. Statistical analysis was performed using Prism 5 (GraphPad Software). P values were considered significant at *P* < 0.05 and highly significant at *P* < 0.01 and *P* < 0.001.

## Results

### MMF Signals Through HCAR2 to Dampen *In-Vitro* Activation of Spleen DC but Not Bone Marrow-Derived DC

**Figure 1:**
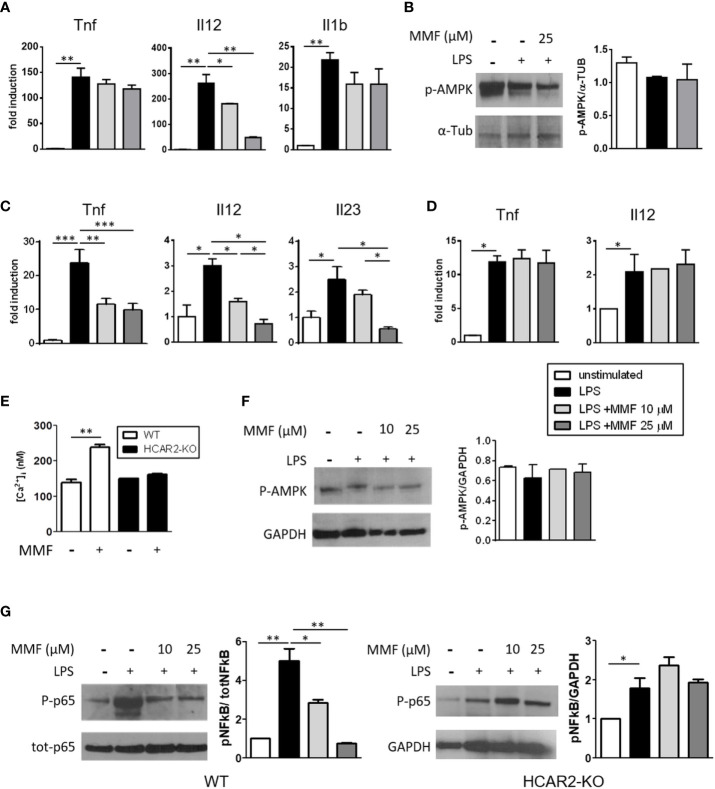
MMF reduces sDC activation through an HCAR2-dependent inhibition of NF-kB p65 phosphorylation. **(A)** MMF significantly decreases *Il12* expression in LPS-activated BM-DC. *Tnf*, *Il12*, and *Il1b* mRNA expression in LPS-activated BM-DC treated or not with MMF for 6 h. **(B)** MMF does not signal through AMPK/Sirt1 axis in activated BM-DC. Western blotting for P-AMPK in LPS-activated BM-DC treated or not with MMF for 30 min. Representative blot (left panel) and densitometric quantification of replicate experiments (right panel). **(C)** Treatment with MMF reduces the pro-inflammatory cytokine profile of LPS-activated sDC. *Tnf*, *Il12*, and *Il23* mRNA expression in LPS-activated sDC treated or not with MMF for 6 h. **(D)** The anti-inflammatory effect of MMF on sDC is dependent on HCAR2. *Tnf* and *Il12* mRNA expression in LPS-activated sDC isolated from HCAR2-KO mice, in presence or absence of MMF for 6 h. **(E)** MMF induces HCAR2-dependent increase in [Ca2+]i in sDC. [Ca2+]i measured by Fura 2AM analysis in sDC from WT and HCAR2-KO mice treated or not with MMF (25 μM) for 1 h. **(F)** MMF signaling through HCAR2 in LPS-activated sDC does not involve AMPK/Sirt1 axis. Western blotting for P-AMPK in LPS-activated sDC treated or not with MMF for 30 min. Representative blot and densitometric quantification of replicate experiments are shown (left and right panels, respectively). **(G)** MMF signaling through HCAR2 in LPS-activated sDC inhibits NF-kB phosphorylation. Western blotting for P-p65 and tot-p65 in LPS-activated sDC from WT (left panel) and HCAR2-KO (right panel) mice treated or not with MMF for 30 min. Representative blots and densitometric quantification of replicate experiments are shown. Real time PCR data are presented as fold induction of gene expression in treated cells over untreated cells. Quantification of Western blotting by densitometric analysis of bands is presented as P-AMPK over α-Tub **(B)** or P-p65 over tot-p65, normalized to GAPDH **(F)**. α-Tub **(B)** or GAPDH **(F, G)** were assessed as loading controls. Results are shown as mean ± SEM of at least N = 3 experiments. *P < 0.05, **P < 0.01, and ***P < 0.001.

DMF/MMF exert their anti-inflammatory action on activated human and mouse DC at least partially through inhibition of NF-kB activation (5, 6, 29). We postulated that signaling through HCAR2 *via* the AMPK/Sirt1 axis could be involved in this mode of action, as it is in mediating the anti-inflammatory effect of MMF on microglia (8). We first confirmed the anti-inflammatory effect of MMF on DC by measuring the mRNA expression of cytokines commensurate with DC activation, in BM-DC that had been stimulated with LPS in the presence of MMF. Exposure to MMF decreased the expression of *Il12* by activated BM-DC (**Figure 1A**), corroborating the data of Peng et al. (6), in a dose-dependent manner, but had little or no effect on their expression of *Tnf* and *Il1b*. To assess the possible involvement of the AMPK/Sirt1 axis in downregulating the pro-inflammatory phenotype of activated BM-DC, we analyzed the phosphorylation state of AMPK, which represents a salient step of the pathway, in LPS-activated BM-DC treated or not with MMF. p-AMPK did not increase in the treated cells (**Figure 1B**), indicating that the AMPK/Sirt1 axis is not involved in the MMF-mediated downregulation of *Il12* in activated BM-DC. Because, in contrast to culture-expanded BM-DC, sDC are more relevant to our study and represent DC that would be exposed to MMF *in vivo*, we investigated the effect of MMF on freshly isolated LPS-activated sDC and found that it exerted a strong, dose-dependent anti-inflammatory effect on these cells, significantly decreasing their mRNA expression of *Tnf*, *Il12*, and *Il23* (**Figure 1C**). To determine the possible involvement of HCAR2 triggering in the effect of MMF on sDC, we analyzed the expression of the same cytokines in sDC isolated from HCAR2-KO mice and treated with LPS and MMF in the same way. MMF had no effect on the expression of the pro-inflammatory cytokines by the HCAR2-KO cells (**Figure 1D**), supporting the possibility that, in contrast to what we observed with BM-DC, MMF signals through HCAR2 in sDC. We corroborated these data by the demonstration that the differential effect of MMF on BM-DC and sDC could be related to a difference in expression of HCAR2 on these cells. Indeed, we found that the expression of HCAR2 at both mRNA and protein levels is lower in BM-DC than in sDC (**Supplementary Figure 2**). Because activation of HCAR2 in immune cells results in an increase in Ca^2+^ as second messenger (30), we measured the intracellular Ca^2+^ concentration in sDC from WT and HCAR2-KO mice upon *in-vitro* exposure to MMF. The intracellular Ca^2+^ concentration was indeed elevated in WT sDC upon exposure to MMF, but such an increase was not observed with sDC derived from HCAR2-KO mice (**Figure 1E**), corroborating our contention that signaling through HCAR2 plays an important role in the effect of MMF on sDC. To verify if, in these cells, MMF signaling through HCAR2 involves the activation of the AMPK/Sirt1 anti-inflammatory pathway, as occurs in microglia (8), we assessed the phosphorylation status of AMPK in activated sDC exposed or not to MMF, by Western blotting. Exposure to MMF did not affect the phosphorylation state of AMPK in sDC (**Figure 1F**), suggesting that its effect is not mediated *via* the AMPK/Sirt1 axis. In contrast, we found that MMF inhibited the NF-kB pathway in WT sDC by reducing the LPS-induced hyperphosphorylation of the p65 subunit in these cells (**Figure 1G**, left panel); this effect was not observed in HCAR2-KO sDC (**Figure 1G**, right panel), confirming its dependence on HCAR2 signaling.

Altogether, these results indicate that the HCAR2-mediated inhibition of pro-inflammatory cytokine expression by MMF can occur through different signaling axes in different cell types (e.g. *via* inhibition of NF-kB phosphorylation in sDC, or *via* activation of AMPK-Sirt1 pathway, and thereby inhibition of NF-kB activity, in microglia). Moreover, the anti-inflammatory effect of MMF mediated by HCAR2 also appears to be dependent on the differentiation status and/or origin of the cell (e.g. bone marrow-derived DC vs. sDC).

### *In Vitro*, MMF Signaling Through HCAR2 Affects Resting and Activated IEC Differentially

**Figure 2:**
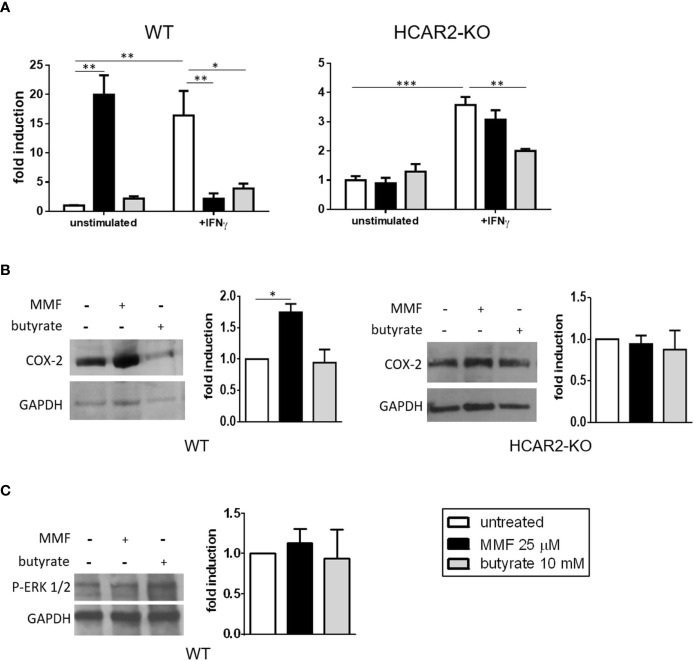
Under non-inflammatory conditions, MMF signals through the HCAR2/COX-2 pathway to increase the mRNA expression of the pro-inflammatory *Tnf* in IEC. **(A)** MMF differentially affects *Tnf* expression in IEC under different conditions of activation. *Tnf* mRNA expression in IEC isolated from WT (left panel) or HCAR2-KO (right panel) mice and activated or not with IFNγ (100 ng/ml), in presence or absence of MMF or butyrate for 6 h. **(B)** MMF, but not butyrate, induces COX-2 in unstimulated IEC, *via* HCAR2. Western blotting for COX-2 in IEC isolated from WT (left panels) and HCAR2-KO (right panels) mice treated or not with MMF or butyrate for 6 h. **(C)** MMF does not activate HCAR2/ERK1/2 pathway in unstimulated IEC. Western blotting for P-ERK1/2 in IEC isolated from WT mice treated or not with MMF or butyrate for 6 h. Representative blots and densitometric quantification graphs are shown. Real-time PCR data are presented as fold induction over the expression of the gene in untreated cells. GAPDH was assessed as loading control for Western blotting and quantification by densitometric analysis of bands is presented as COX-2 over GAPDH **(B)** or p-ERK1/2 over GAPDH **(C)**. All results are shown as mean ± SEM of at least N = 3 experiments. *P < 0.05, **P < 0.01, and ***P < 0.001.

In keratinocytes, HCAR2 ligands can trigger the activation of pro-inflammatory pathways (i.e. the HCAR2/COX-2 and the HCAR2/ERK1/2 MAPK), which results in skin flushing (10, 11). We have speculated that such a pro-inflammatory effect could underlie the gastrointestinal side effects associated with DMF therapy (13). To assess such a possibility, we first confirmed, using immunofluorescence microscopy analysis, that HCAR2 is indeed expressed by *in-vitro*-cultured IEC (**Supplementary Figure 3**). We then tested the nature of MMF effect on naïve IEC under resting (unstimulated) and inflammatory (stimulation with IFNγ) conditions. Exposure of unstimulated IEC to MMF had a pro-inflammatory effect, greatly increasing their *Tnf* expression; in contrast, in IFNγ-stimulated IEC, MMF exerted an anti-inflammatory effect, reverting the increased expression of *Tnf* to basal levels (**Figure 2A**, left panel). The same experiment performed with IEC isolated from HCAR2-KO mice revealed that the contrasting effects of MMF were dependent on HCAR2 signaling. Indeed, in the absence of the receptor, MMF had no effect on unstimulated IEC, nor did it induce reversal of IFNγ-mediated *Tnf* upregulation (**Figure 2A**, right panel). Overall, these data suggest that MMF signaling through HCAR2 is modulated by the *in-vitro* experimental conditions of the cells. In parallel, to understand if the effects mediated by HCAR2 signaling are specific for MMF, or could also be triggered by other HCAR2 agonists, we treated IEC (both in resting and inflammatory conditions) with butyrate, a bacterial metabolite that is an endogenous ligand of HCAR2 and can exert an anti-inflammatory effect by inhibiting NF-kB activation in intestinal cells (31). Our data show that, in contrast to what occurred with MMF, exposure of unstimulated IEC to butyrate did not affect *Tnf* expression (**Figure 2A**, left panel), whereas under inflammatory conditions butyrate exerted an anti-inflammatory effect (**Figure 2A**, left panel), which was independent of HCAR2 (**Figure 2A**, right panel).

To ascertain if the pro-inflammatory effect of MMF in resting IEC could be related to the activation of the HCAR2/COX-2 pathway, as observed with skin flushing under DMF treatment (10), we measured their expression of COX-2, the enzyme that catalyzes the synthesis of prostaglandins and is induced upon activation of the pathway (32). We found that treatment of unstimulated WT IEC with MMF, but not with butyrate, led to an increase in their level of COX-2 (**Figure 2B**, left panels), strongly suggesting that the pro-inflammatory effect of MMF on unstimulated WT IEC is mediated through the COX-2/prostaglandin pathway. To ascertain that HCAR2 signaling is responsible for MMF activation of the prostaglandin pathway in these cells, we measured the expression of COX-2 in MMF-treated IEC isolated from HCAR2-KO mice. The expression of COX-2 in these cells did not differ from that of non-MMF-treated HCAR2-KO IEC (**Figure 2B**, right panels), demonstrating that the activation of the COX-2/prostaglandin pathway by MMF is indeed HCAR2-dependent. In addition, we excluded the possibility that the effect could be related to the activation of HCAR2/ERK1/2 pathway: there was no change in the phosphorylation status of ERK 1/2 upon exposure to MMF or to butyrate (**Figure 2C**).

These data suggest that the prostaglandin pathway is involved in the HCAR2-dependent pro-inflammatory effect exerted by MMF *in vitro* on unstimulated IEC.

### *In Vivo*, DMF Treatment of EAE-Affected Mice Exerts a Pro-Inflammatory Effect on IEC That Is Mediated Through the HCAR2/ERK1/2 Pathway

**Figure 3:**
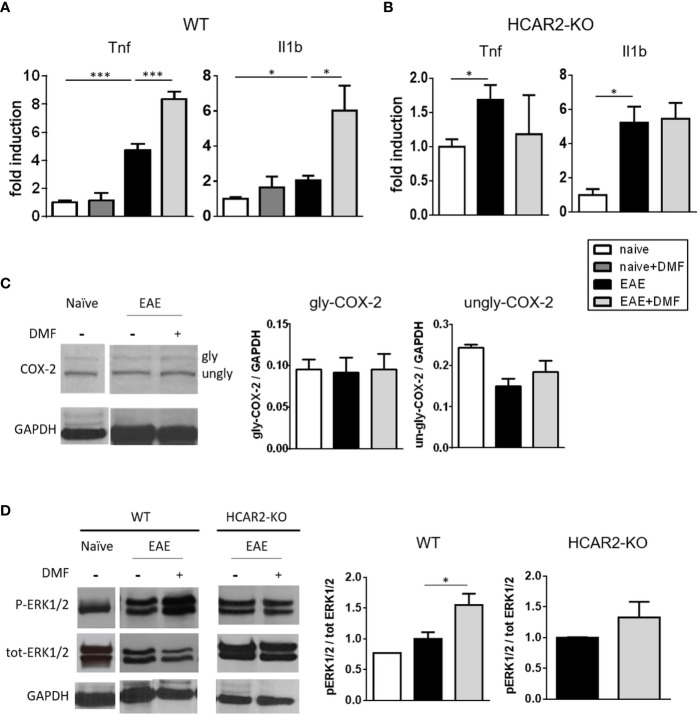
*In vivo* DMF treatment of EAE-affected mice exerts a pro-inflammatory effect on IEC that is dependent on the activation of HCAR2/ERK1/2 pathway. **(A, B)** *In-vivo* DMF treatment of EAE-affected mice results in increased expression of pro-inflammatory cytokines by IEC, which is HCAR2-dependent. *Tnf* and *Il1b* mRNA expression in IEC isolated from naïve and EAE-affected WT **(A)** or HCAR2-KO **(B)** mice, that were treated or not with DMF. Data are presented as fold induction of gene expression in cells isolated from EAE-affected mice (treated or not with DMF) over cells isolated from naïve mice. **(C, D)** In EAE, the pro-inflammatory effect of DMF on IEC involves the HCAR2/ERK1/2 pathway, rather than the HCAR2/COX-2 pathway. Western blotting for COX-2 **(C)** and ERK1/2 **(D)** in IEC from naïve and EAE-affected WT **(C, D)** and HCAR2-KO **(D)** mice that were treated or not with DMF. GAPDH was assessed as loading control. One representative blot is shown and quantification by densitometric analysis of bands is presented as gly- or ungly-COX-2 over GAPDH **(C)**, or P-ERK1/2 over tot-ERK1/2, normalized to GAPDH **(D)**. Average clinical scores of mice at the time of the analysis are: WT-EAE-affected mice: 3 ± 0.17 (SEM); WT-EAE-affected mice treated with DMF: 2.75 ± 0.11; HCAR2-KO-EAE-affected mice: 3.1 ± 0.24; HCAR2-KO-EAE-affected mice treated with DMF: 3.05 ± 0.27. Results are shown as mean ± SEM of at least N = 3 experiments with at least N = 3 mice per group per experiment. *P < 0.05 and ***P < 0.001.

To determine if the pro-inflammatory action of MMF on unstimulated IEC translates *in vivo*, we first treated naïve mice with DMF by daily oral gavage for 4 days and analyzed the expression of *Tnf* and *Il1b* in isolated IEC. In contrast to what we observed in the in-vitro experiments with resting IEC exposed to DMF, the expression of pro-inflammatory cytokines was not increased in IEC isolated from DMF-treated naïve mice (**Figure 3A**). In EAE-affected mice, IEC displayed an increased mRNA expression of *Tnf* and *Il1b* (**Figure 3A**), on par with the observation that EAE is associated with an inflamed gut at the early phase of disease (33), and treatment of the EAE-affected mice with DMF by daily oral gavage for 4 days from disease onset further increased their inflammatory profile (**Figure 3A**). In contrast, *Tnf* and *Il1b* expression by IEC in EAE-affected HCAR2-KO mice was not affected by treatment with DMF (**Figure 3B**), implicating HCAR2 signaling in this pro-inflammatory effect of DMF on IEC in EAE-affected mice.

To assess if the HCAR2/COX-2 pathway is involved in the pro-inflammatory effect of DMF *in vivo*, as it is *in vitro*, we analyzed the expression of COX-2 in IEC isolated from EAE-affected mice treated or not with DMF. Western blotting analysis of *ex-vivo* IEC (**Figure 3C**) revealed the two known forms of COX-2, glycosylated (gly-COX-2, 72-74 kDa) and un-glycosylated (un-gly-COX-2, 66-70 kDa). It is unclear why both forms of COX-2 are detected in the *ex-vivo* cells, whereas only the higher form was observed in the *in-vitro* experiment, as the Western blotting analyses were performed according to the same protocol. One possible explanation would be that the culture conditions essential for the growth of the IEC *in vitro* might themselves induce activation of the cells (34), leading to an increase in glycosylation of COX-2. We quantified gly-COX-2 since it is considered the active form of the enzyme (35), and our data indicate that DMF treatment had no effect on its expression (**Figure 3C**), suggesting that, in contrast to what occurred *in vitro* in unstimulated IEC, the pro-inflammatory effect of DMF *in vivo* in EAE-affected mice is not mediated through activation of the HCAR2/COX-2 pathway. Since HCAR2 can also signal through the activation of the pro-inflammatory ERK MAPK pathway (HCAR2/ERK1/2 pathway) (11), we speculated that activation of this pathway could be responsible for the pro-inflammatory effect of DMF *in vivo*; we therefore monitored the phosphorylation of ERK1/2 in IEC isolated from EAE-affected mice treated or not with DMF. Western blotting results show that, indeed, treatment with DMF led to increased phosphorylation of ERK1/2 in these cells (**Figure 3D**), and that it involves HCAR2 signaling as such an effect was not seen in *ex-vivo* IEC from EAE-affected HCAR2-KO mice (**Figure 3D**). These findings demonstrate that, also in different experimental settings, and thereby under different environmental conditions, MMF can signal through HCAR2 *via* different pathways to exert different effects.

### DMF Treatment Does Not Worsen EAE-Associated Morphological Alterations of the Gut

**Figure 4:**
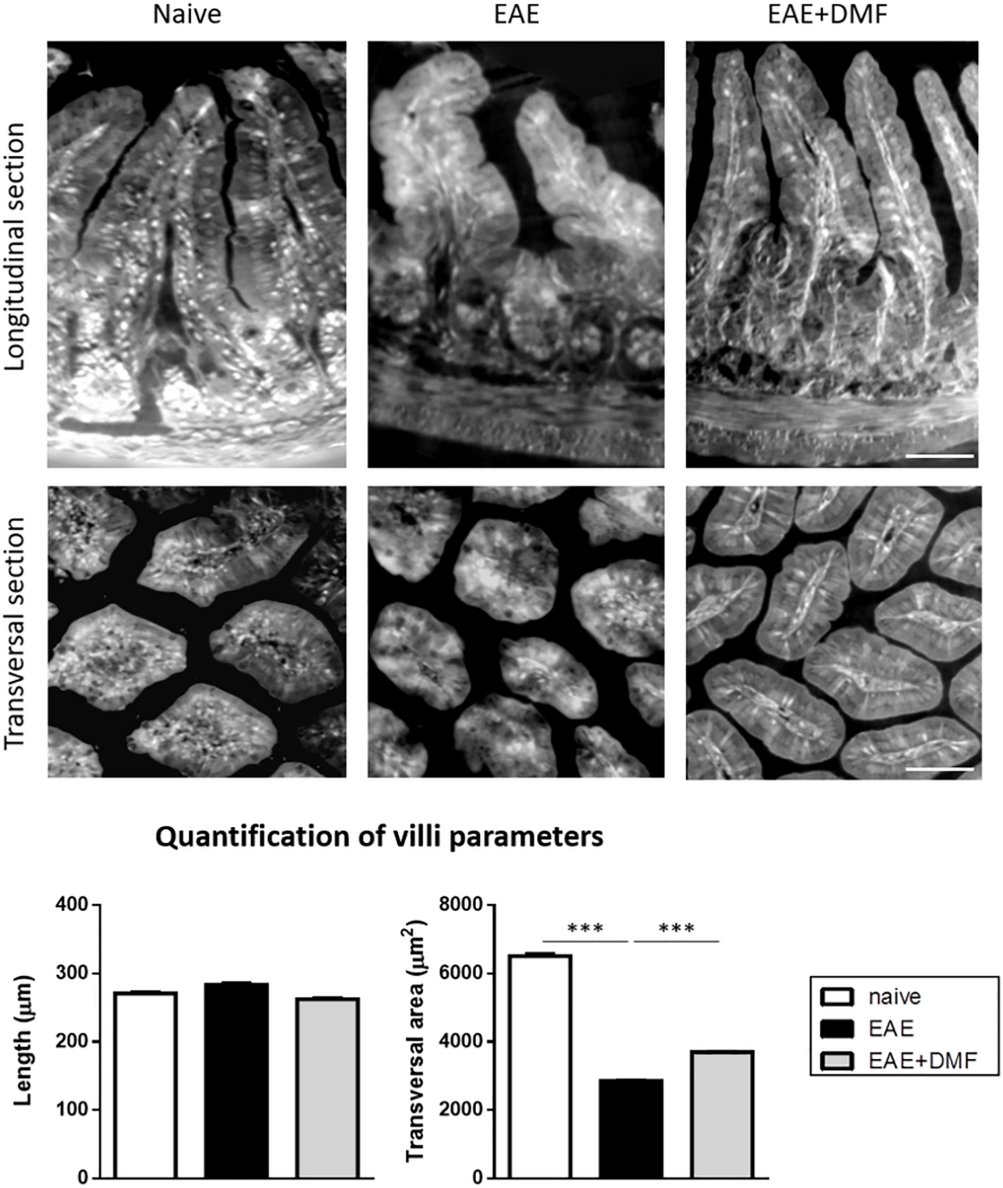
DMF treatment does not worsen the morphological alterations induced by EAE at the level of the ileum. Ilea were isolated from naïve mice and at 4 d post onset from EAE-affected mice treated or not with DMF. Top panels show representative XPCT images (longitudinal and transversal sections) of ileum in naïve, EAE-affected, and EAE-affected DMF-treated mice. Bottom panels show the quantification of the morphological parameters (length and transversal area) performed using ImageJ software (as described in *Methods*) for at least 120 villi/mouse (N = 3 mice per condition). Average clinical scores of mice at time of the analysis: WT-EAE-affected mice: 3 ± 0.17; WT-EAE-affected mice treated with DMF: 2.75 ± 0.11. Scale bars: 50 μm. Results are shown as mean ± SEM. ***P < 0.001.

Morphological and immunological alterations at the level of the small intestine have been reported to occur in EAE-affected mice (33). Our *ex-vivo* data indicate that treatment of EAE-affected mice with DMF has a pro-inflammatory effect on ileum IEC; we therefore investigated whether or not DMF treatment could worsen EAE-related morphological and immunological alterations of the small intestine, focusing on the ileum as the section of the small intestine seemingly most affected by an inflammatory response (36, 37). To gain a comprehensive view of ileum morphology, we used XPCT, which allows 3D visualization of tissue structure without additional staining (**Figure 4**) (38). The length and the transversal section area of the villi in the ileum of naïve and EAE-affected mice, treated or not with DMF for 4 days from disease onset, were measured on the images obtained. Analysis of the data indicated that, at this acute phase of EAE, ileum villi did not show a significant alteration in length, but displayed a significantly smaller (by about 56%) transversal area at the base of the villus (**Figure 4**). DMF treatment partially restored such alteration (by about 29%; **Figure 4**).

### Treatment With DMF Affects the Immunological Profile of Ileum and Mesenteric Lymph Nodes in EAE-Affected Mice

**Figure 5:**
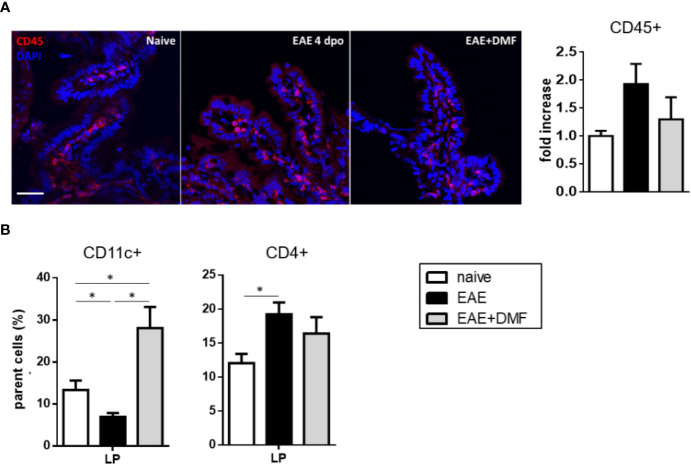
DMF treatment differentially affects the infiltrated immune cells in ileum LP of EAE-affected mice. **(A)** DMF treatment does not significantly revert the increase in CD45+ cells in ileum LP induced by EAE. Confocal microscopy images of the ileum of naïve and EAE-affected mice treated or not with DMF (left panel) and quantification of the CD45+ cells (right panel). Slides were stained with anti-CD45 antibody (red) and with DAPI (blue). Scale bar: 50 μm. **(B)** DMF treatment of EAE-affected mice increases the percentage of CD11c+ cells, but has no effect on the increased CD4+ cell population in the ileum LP. FACS analysis of CD11c+ and CD4+ T cells in LP from naïve mice and at 4 days post onset from EAE-affected mice, treated or not with DMF. Average clinical scores of mice at time of the analysis: WT-EAE-affected mice: 3 ± 0.17; WT-EAE-affected mice treated with DMF: 2.75 ± 0.11. Results are shown as mean ± SEM of at least N = 3 independent experiments, with at least N = 3 mice per group per experiment. *P < 0.05.

To further assess possible immune-related inflammation and the effect of DMF treatment on EAE gut, we analyzed immune cell infiltration at the level of the ileum LP of naïve and EAE-affected mice, treated or not with DMF, by immunofluorescence with anti-CD45 antibody, to detect circulating leukocytes. As our data show some increase in CD45+ cells, albeit not reaching significance, in the ileum LP of EAE-affected mice as compared to naïve mice (**Figure 5A**), we measured in the LP the percentage of possible infiltrating immune cell populations, such as CD11c+ DC, for their role as antigen-presenting cells, and CD4+ cells, which represent the T-cell subset most abundant in the LP (39). Our data revealed a decrease in CD11c+ cells and an increase in CD4+ cells in EAE-affected mice (**Figure 5B**). DMF treatment of EAE-affected mice reverted the frequency of CD11c+ cells to above that of naïve mice, but had no effect on that of CD4+ cells (**Figure 5B**).

**Figure 6:**
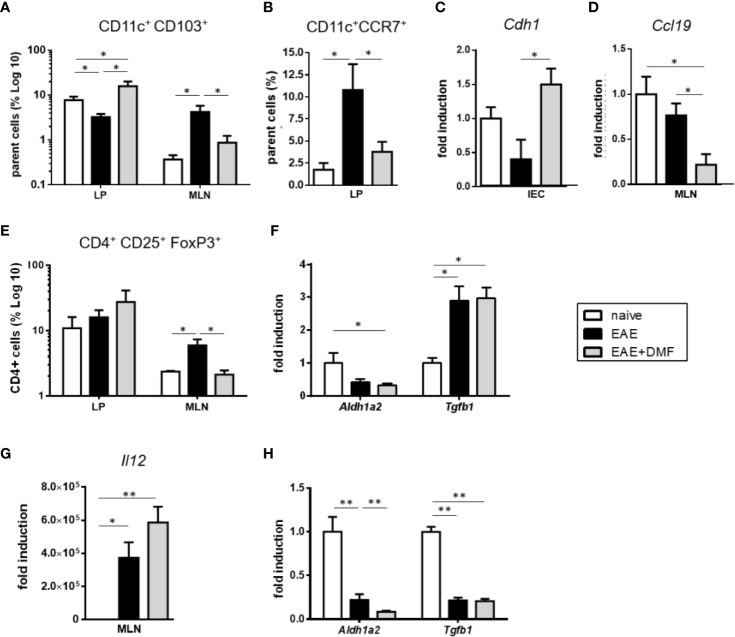
DMF treatment likely affects both migration and differentiation of immune cells in the small intestine of EAE-affected mice. **(A)** CD11c+CD103+ cells are retained in LP of EAE-affected DMF-treated mice. FACS analysis of CD11c+CD103+ cells in the parent cell population (i.e. identified as non IEC by size and granularity) in ileum LP and MLN of naïve and EAE-affected mice, treated or not with DMF. **(B)** DMF treatment of EAE-affected mice decreases the percentage of migratory CD11c+CCR7+ cells in ileum LP. FACS analysis of CD11c+CCR7+ cells over parent cells (as above) in ileum LP of naïve and EAE-affected mice, treated or not with DMF. **(C)** The expression of the CD103 ligand, *Cdh1*, is increased in IEC of EAE-affected DMF-treated mice. *Cdh1* mRNA expression in IEC from naïve and EAE-affected mice treated or not with DMF. **(D)** The expression of the CCR7 ligand, *Ccl19*, is decreased in IEC of EAE-affected DMF-treated mice. *Ccl19* mRNA expression in IEC from naïve and EAE-affected mice treated or not with DMF. **(E)** The pattern of Treg cell frequencies of naïve and EAE-affected mice treated or not with DMF parallels that of CD11c+CD103+cells in MLN, but not in LP. FACS analysis of CD4+CD25+FoxP3+ cells, within the CD4+ cell population, in ileum LP and in MLN from naïve and EAE-affected mice, treated or not with DMF. **(F)** DMF treatment does not revert the EAE-induced decrease in *Aldh1a2* or increase in *Tgfb1* in MLN. *Aldh1a2* and *Tgfb1* mRNA expression in MLN cells from naïve and EAE-affected mice treated or not with DMF. **(G)** DMF treatment does not revert the increased *Il12* expression induced by EAE in MLN. *Il12* mRNA expression in MLN isolated from naïve and EAE-affected mice treated or not with DMF. **(H)** DMF treatment of EAE-affected mice further decreases *Aldh1a2* expression in IEC but does not revert that of *Tgfb1*. *Aldh1a2* and *Tgfb1* mRNA expression in IEC isolated from naïve and EAE-affected mice treated or not with DMF. Average clinical scores of mice at the time of the analysis: WT-EAE-affected mice: 3 ± 0.17; WT-EAE-affected mice treated with DMF: 2.75 ± 0.11. Real time PCR data are presented as fold induction of gene expression in cells from EAE-affected mice (treated or not with DMF) over cells from naïve mice. FACS and PCR results are shown as mean ± SEM of at least N = 3 experiments, with at least N = 5 mice per group per experiment. *P < 0.05 and ** *P* < 0.01.

In mice, most of the intestinal DC are characterized by their surface expression of CD11c and CD103 (40, 41), and intestinal CD11c+CD103+ DC are involved in the induction of tolerogenic immune responses (41, 42). Thus, in view of the increased frequency of CD11c+ cells in the LP of EAE-affected mice upon DMF treatment and to further understand how such a treatment might impact the immunological profile of the gut, we evaluated the percentage of immune cells in tissues that are relevant for intestinal immunity, LP and MLN. Indeed, in steady state conditions, CD11c+CD103+ cells actively migrate from the LP to the MLN, where they drive regulatory T (Treg)-cell differentiation by inducing the expression of FoxP3 in naive CD4+ T cells (42). We therefore assessed these two populations in LP and MLN of EAE-affected mice treated or not with DMF, as compared to naive mice. As can be seen in **Figure 6A**, the frequency patterns of CD11c+CD103+ cells in LP under the different conditions were the reverse of those in MLN. Thus, as shown in **Figure 6A**, in cells isolated from LP of EAE-affected mice the frequency of CD11c+CD103+ DC was reduced by more than 50% that of naïve mice, but was fully reverted and enhanced by DMF treatment (**Figure 6A**). In contrast, in cells isolated from MLN of EAE-affected mice, the frequency of CD11c+CD103+ DC was increased, but reverted to basal levels upon DMF treatment (**Figure 6A**). These data suggest that in EAE-affected mice, CD11c+CD103+ cells might actively migrate from the LP to MLN, as observed in chronic ileitis (43) and upon Salmonella infection (44), but are retained in the LP upon DMF treatment. To ascertain such a possibility, we analyzed the expression of C-C chemokine receptor type 7 (CCR7), a receptor involved in DC migration towards MLN (45), on CD11c+ cells, of which the majority that migrate to MLN are CD103+ (42, 46). Flow cytometry data indicated that CD11c+CCR7+ cells were increased in the ileum LP of EAE-affected mice as compared to naïve mice (**Figure 6B**); DMF treatment abrogated such an increase (**Figure 6B**), suggesting that it might affects the migration of the CD11c+CD103+ cells from LP to MLN. To address this possibility, we analyzed if DMF treatment affected IEC expression of E-cadherin (Cdh1), the ligand of CD103 that is involved in the retention of DC within the LP (47). Indeed, DMF treatment of EAE-affected mice significantly increased the expression of Cdh1 in isolated IEC (**Figure 6C**). To assess whether or not the retention of CD11c+CD103+ DC could also be associated with a reduced migration of the cells towards the CCR7 ligand, chemokine (C–C motif) ligand 19 (CCL19) that is constitutively expressed in lymph nodes (48), we tested the levels of Ccl19 in MLN. There was no difference in Ccl19 expression in MLN from naïve mice vs EAE-affected mice; however, DMF treatment was associated with a significantly decreased expression of Ccl19 (**Figure 6D**). Altogether, these data corroborate our speculation that DMF induces a retention of CD11c+CD103+ DC in the LP through reduced migration (fewer CD11c+CCR7+ cells) and increased expression of Cdh1 by IEC, together with the reduced levels of the attracting chemokine Ccl19 in the MLN (**Figures 6A–D**). Because CD11c+CD103+ cells drive Treg-cell differentiation and imprint gut-homing phenotype on these cells (41, 42), we examined the percentage of CD4+CD25+FoxP3+ Treg cells both in LP and in MLN. In MLN, the pattern of Treg cells under these conditions (**Figure 6E**) paralleled that observed for CD11c+CD103+ cells (**Figure 6A**). Thus, in EAE-affected mice, we observed an increase in Treg cells in the MLN, as compared to that in naïve mice; unexpectedly, we did not observe a corresponding increase in Treg-cell frequency in the LP (**Figure 6E**). Homing of Treg cells to LP is dependent upon the secretion of retinoic acid (RA), and their differentiation to Treg cells necessitates their exposure to transforming growth factor β (TGFβ) (49, 50). We speculated that the unchanged frequency of Treg cells in the LP of EAE-affected mice as compared to naive mice might be related to a decrease in RA in the MLN. We therefore analyzed the mRNA expression of aldehyde dehydrogenase (Aldh1a2), the enzyme involved in the synthesis of RA. Aldh1a2 expression was indeed decreased in MLN of EAE-affected mice (**Figure 6F**). As exposure to RA induces the expression of CCR9, which imparts gut-homing functionality to T cells (41), we would expect it to be reduced in MLN of EAE-affected mice, and indeed, we could not detect any Ccr9 expression in the MLN in EAE (data not shown). Tgfb expression was however increased in the MLN in these mice, on par with the increased frequency of Treg cells (**Figure 6F**). In contrast, upon DMF treatment of EAE-affected mice, the frequency of Treg cells in MLN had reverted to that of naïve mice (**Figure 6E**), in parallel with the frequency of CD11c+CD103+ cells (**Figure 6A**). As observed in LP of EAE-affected mice, there was no change in Treg cells in LP of the DMF-treated mice (**Figure 6E**), suggesting that the decrease in Treg cells in the MLN under DMF treatment was not related to an increased homing of the cells from the MLN to the LP. Thus, we speculated that the reduced Treg cells in MLN of EAE-affected mice upon DMF treatment might be related to decreased homing and/or decreased differentiation of T cells to Treg cells. Aldh1a2 expression in MLN in EAE-affected mice was also reduced upon DMF treatment, as compared to naïve mice (**Figure 6F**), and Ccr9 expression was undetectable (data not shown). In contrast, *Tgfb* expression in EAE-affected DMF-treated mice was significantly increased as compared to that in naïve mice and did not differ from that of untreated mice (**Figure 6F**). As Th1 cell-promoting cytokines have been shown to reduce TGF-β-mediated induction of Treg cells (51), we assessed the expression of *Il12*, the typical Th1 cell-promoting cytokine expressed by activated DC, in MLN. We found that *Il12* expression was indeed greatly increased in the MLN of EAE-affected mice treated or not with DMF (**Figure 6G**), suggesting that at least some of the CD11c+CD103+ cells might have reverted to a pro-inflammatory profile (52).

In view of the increased frequency of CD11c+CD103+ DC in the LP under DMF treatment (**Figure 6A**), we would have expected a parallel increase in Treg cells in this tissue. In addition to the possible reduced homing of Treg cells from MLN to LP in EAE-affected mice treated with DMF, the unchanged frequency of these cells in LP could also be related to the inflammatory environment of the LP in these mice (**Figure 3A**). In the LP, CD103+ DC are “educated” by IEC to promote Treg-cell differentiation through IEC secretion of RA and TGFβ (41, 53). Accordingly, we assessed the mRNA expression of *Aldh1a2* and *Tgfb1* in IEC and observed a strong downregulation of these genes in IEC isolated from EAE-affected mice treated or not with DMF in comparison to IEC from naïve mice (**Figure 6H**), suggesting that IEC under these inflammatory conditions have a reduced capacity to promote tolerogenic DC and therefore Treg-cell differentiation, providing another possible explanation for the unchanged Treg-cell frequency in the LP of these mice.

Together with data presented in **Figure 3**, these results suggest that, in complete contrast to what we observed *in vitro* in IEC under inflammatory conditions (**Figure 2A**), DMF treatment does not alleviate gut inflammation in EAE.

## Discussion

**Figure 7:**
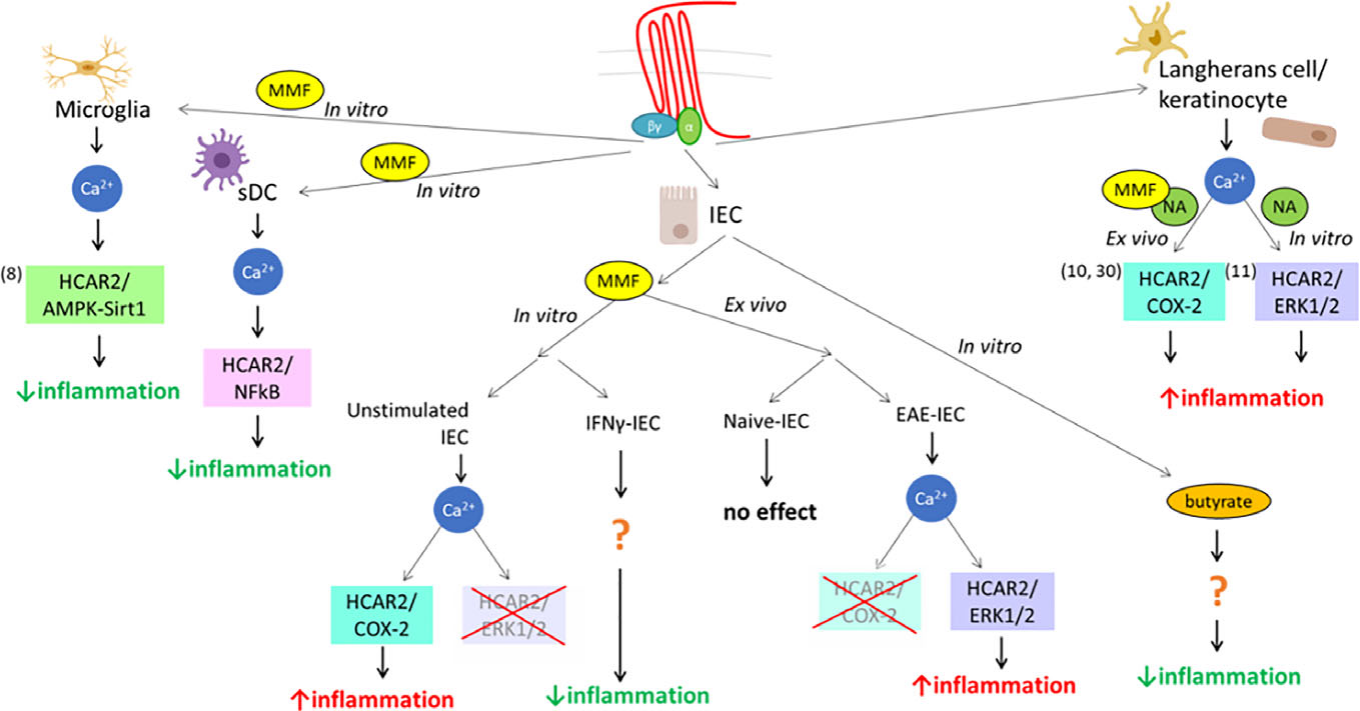
Scheme representing the cell, ligand, and environmental biases of HCAR2 signaling. This scheme summarizes the various biases of the pleiotropically linked receptor for MMF, HCAR2, at system and ligand levels, from data obtained in this and previous studies. It shows the usage of three different pathways used in three different cell types (microglia, DC, and IEC) *in vitro* upon triggering of HCAR2 by MMF, with different outcomes (decrease or increase of inflammatory profile of the cell) associated with the different pathways; the impact of the environment (*in vitro* vs *in vivo*, under resting or inflammatory conditions) on MMF signaling; and the differential outcome/signaling by different ligands (MMF, NA, butyrate).

MMF, the metabolite of DMF, is an exogenous ligand for HCAR2, and we previously demonstrated that it can exert its anti-inflammatory action through the AMPK/Sirt1 pathway (8). However, DMF treatment in patients with MS is also associated with pro-inflammatory side effects, the most prominent of which are skin flushing and symptoms reflecting gut inflammation (54), suggesting that it could also signal through pro-inflammatory pathways in different cell types. In this study, we demonstrate that signaling of MMF through HCAR2 can indeed be mediated through the activation of different pathways that have distinct, or even opposite, effects on cell function, depending on the context, that is cell type/origin/differentiation and experimental/environmental conditions. This phenomenon, called “biased signaling”, has been shown for other seven-transmembrane G protein-coupled receptors and occurs when the activation of a receptor triggers a specific signaling pathway at the expense of other(s) (55), under defined conditions. In *in-vitro* DC cultures, MMF had a very different effect depending on the origin of the cells and their maturation, downregulating only the expression of *Il12* in BM-DC, but of all three pro-inflammatory cytokines tested in sDC. These findings are reminiscent of what has been observed in immature vs mature human neutrophils treated with another HCAR2 ligand, NA, which induced apoptosis in mature blood neutrophils with detectable levels of HCAR2, but not in immature neutrophils isolated from the bone marrow, which do not express the receptor (56). The difference is therefore likely due to the greater levels of HCAR2 expression in sDC, as variation in receptor levels is a well-known contributor to heterogeneity in ligand signaling (55, 57). In contrast to what occurs in microglia (8), HCAR2-dependent MMF effect on pro-inflammatory cytokine expression in sDC was mediated through inhibition of NF-kB, but not *via* AMPK/Sirt1 signaling (see **Figure 7**). These data support our contention that the HCAR2-dependent MMF signal can be mediated *via* different pathways in cells of different types and/or origins for a similar anti-inflammatory outcome, reflecting a cell-background-dependent, or so-called “system”, bias (55) of MMF signaling *via* HCAR2. In the same context of system bias, MMF signaling in unstimulated “resting” IEC exerted a pro-inflammatory HCAR2-dependent effect through activation of the COX-2 pathway; a similar outcome of MMF exposure dependent on HCAR2 is observed in keratinocytes, which underlies the second phase of the flushing reaction, a typical side effect of DMF therapy (10). Indeed, this latter study prompted our analysis of the effect of MMF on IEC, based on the postulate that triggering of inflammation in the gut through MMF signaling *via* the HCAR2/COX-2 pathway could also be responsible for the gastro-intestinal inflammation occurring in the initial phase of DMF therapy in MS patients (13, 54). However, the HCAR2-dependent effect of MMF *in vitro* in cultured IEC and *ex vivo* in IEC isolated from EAE-affected mice also displayed environment-related bias (58). Thus, while, *in vitro*, exposure to MMF had a pro-inflammatory effect in resting IEC and an anti-inflammatory effect in IFNγ-stimulated IEC, we observed an opposite effect in *ex-vivo* IEC isolated from DMF-treated mice. Indeed, DMF had no effect on IEC in treated naïve mice but exerted a pro-inflammatory effect on IEC in treated EAE-affected mice, that is under pro-inflammatory conditions. HCAR2-dependent pathways activated upon exposure to fumarate also differed, with *in-vitro* exposure to MMF inducing inflammation *via* the HCAR2/COX-2 pathway in unstimulated IEC, whereas activation of the HCAR2/ERK1/2 pathway, which we excluded in our *in-vitro* study, underlay the increase in the inflammatory status of IEC induced by DMF treatment *in vivo* in EAE-affected mice (see **Figure 7**). Thus, while our *in-vitro* data apparently concurred with our postulate of MMF signaling through the HCAR2/COX-2 pathway, *ex-vivo* data instead suggest that this is not the case. In support of these findings, a clinical trial aimed at evaluating whether or not administration of a cyclooxygenase inhibitor, acetylsalicylic acid, concomitantly with DMF could alleviate skin flushing and gastrointestinal side effects has demonstrated that, while COX inhibition reduces the incidence and intensity of flushing, it has no impact on gastrointestinal events (59). Such a pathology-driven dynamic bias could have implications in the eventual development of second generation DMF-like drugs devoid of side effects, and further points to the need for *in-vivo* models for their selection (58). However, the novel second-generation oral fumarate diroximel fumarate (DRF) displays improved gastrointestinal tolerability (60). The mechanisms responsible for this amelioration are unclear, as DRF and DMF produce bioequivalent systemic exposure of MMF, but it has been proposed that the potential mechanism of gastrointestinal irritation due to locally elevated levels of methanol derived from DMF metabolism could be considerably mitigated by the use of DRF, the metabolism of which generates a less irritating promoiety, 2-hydroxyethyl succinimide (60). Although a reduced local concentration of methanol within the small intestine may indeed underlie the improved tolerability of DRF, we suggest, based on our findings of biased signaling through HCAR2, that such a beneficial effect could also involve a possible interaction of the minor metabolite of DRF, RCD-8439, with HCAR2, signaling through an as yet undefined anti-inflammatory pathway, as per an agonist bias effect (58, 61) seen with other HCAR2 ligands (**Figure 7**). In this context, several lines of evidence show that the anti-inflammatory effect mediated by HCAR2 signaling is also elicited by other ligands, such as NA and the commensal metabolite butyrate, resulting in decreased macrophage activation and colonic inflammation (62, 63). Gut inflammation leading to intestinal barrier dysfunction has been reported in MS (15, 64) and in EAE (33), albeit as yet poorly described. Its possible consequences are highly important for the possible understanding of the disease etiology and/or progression, and it is of interest in this context to note that there is imaging evidence of demyelination in gastrointestinal disorders associated with intestinal barrier impairment (15). Furthermore, in EAE, improvement of the intestinal barrier function through treatment with a probiotic leads to a decreased infiltration of the CNS by inflammatory T cells, resulting in less severe disease (65). We used XPCT to assess morphological parameters of the ileum in EAE-affected mice at 3D level. Quantification of villus parameters on XPCT images confirmed the 2D histological data obtained for villus length by Nouri et al. (33), who do not observe any alteration in villus length at day 14 after EAE induction, a time point similar to that at which we performed our analysis. Our data, however, demonstrated a significant decrease in the transversal area of the villi of EAE-affected mice. Although Nouri et al. have not assessed the transversal area of the villi, they show other morphological alterations of the ileum with some increase in crypt depth and submucosal thickness (33). These data altogether confirm the disturbing effect of EAE on the small intestine, particularly the ileum, which is a preferential site for inflammation (36, 37). In contrast to what we had hypothesized, DMF treatment did not amplify the morphological alterations observed in EAE-affected mice, as we might have expected from the inflamed profile of *ex-vivo* IEC, nor did it reduce them. There is little information on the effect on intestinal inflammation and barrier dysfunction of disease-modifying drugs for MS and other autoimmune diseases. However, circumstantial evidence obtained with animal models suggest that they could modulate barrier dysfunction and/or the inflammatory response also at gut level (15). Thus, treatment of murine colitis with glatiramer acetate reduced disease expression at clinical and pathological levels, and restored normal levels of syndecan-1 necessary for a stable intestinal epithelial barrier (66); in EAE, teriflunomide induced an increase in MLN CD39+ Treg cells, which, upon transfer in mice at disease onset, reduced clinical severity (67); treatment with DMF alleviated murine colitis, improving intestinal barrier function, and led to a diminution of pro-inflammatory cytokines in the colon (68).

While DMF treatment had no reparative or worsening effect on ileum morphology in EAE-affected mice, it was associated with an increased expression of pro-inflammatory cytokines, suggesting that it might also affect the immune response at gut level. In the LP, CD11c+ DC are functionally divided in two subsets according to their ability to migrate to the MLN and induce the differentiation of T cells; these two subsets are distinguished by the expression of CD103 (41, 46), with CD11c+CD103- cells representing a non-migratory gut-resident population whereas CD11c+CD103+ cells represents the majority of migratory gut-resident DC (46) and their migration from LP to MLN, which occurs constitutively, can be enhanced in inflammatory conditions (43, 44, 69). In this context, CD11c+CD103+ DC appear to play a crucial role in regulating immune responses (47), and therefore inflammation, in the gut. In particular, CD11c+CD103+ DC promote the differentiation of Treg cells, *via* the release of several molecules, in particular RA and TGF-β (70). We have therefore assessed the impact of EAE and DMF treatment on these cells in the LP and MLN of the ileum. Gut CD11c+CD103+ DC have mostly been investigated in mice with colitis, also a T cell-mediated disease, where their frequency is decreased in MLN and small intestinal LP (52), and the impact of DMF treatment on this gut population is not known. We observed a similar decrease in the frequency of CD11c+CD103+ DC in the ileum LP of EAE-affected mice, but their frequency was increased in the MLN. It is therefore likely that the inflammatory environment in the ileum of EAE-affected mice, as reflected by the upregulated expression of pro-inflammatory cytokines by IEC, is responsible for the increased frequency of CD11c+CD103+ DC in MLN that have migrated from the LP, and this possibility is corroborated by the increased frequency in the LP of DC expressing the lymph node-homing marker, CCR7. Indeed, expression of CCR7 by DC is essential for migration and its lack in CCR7-/- mice results in a greatly reduced number of CD103+ DC in the MLN (71). In contrast, under DMF treatment, we observed an opposite pattern of CD11c+CD103+ DC frequencies, which suggested that the treatment induced the retention of the cells in the LP. This possibility was supported by data obtained from tissues reflecting both origin and destination of the cells, with CD103+ DC in MLN representing a population that has migrated from the LP (41). Thus, in the LP, the frequency of CCR7+ CD11c+ cells was reduced and the expression by IEC of the CD103 ligand, the epithelial cell adhesion molecule *Cdh1* (47, 72), was increased, whereas in the MLN, the expression of *Ccl19*, a ligand for CCR7 that induces the migration of DC to lymphoid organs (73), was reduced. As CCL19 induces the maturation of DC with expression of IL-12 and the ensuing induction of inflammatory Th1 cells (74), a decrease in *Il12* in MLN of EAE-affected mice upon DMF treatment could have been expected. While we have at present no clear explanation as to how DMF treatment would maintain an elevated expression of *Il12* in the MLN, it should be noted that DC maturation is not always associated with CCL19 production (75, 76).

IEC induce the development of CD103+ DC responsible for the differentiation of Treg cells through their expression of TGFb and RA (41). Accordingly, we might have expected that the highly increased frequency of CD11c+CD103+ cells we observed in the LP of EAE-affected mice upon treatment with DMF would be reflected in a parallel increase in Treg-cell frequency. Instead, there was no such apparent effect of DMF treatment, which also did not revert the reduction in *Aldh1a2* and *Tgfb1* expression induced in IEC by EAE. We propose two possible, non mutually exclusive explanations: the lack of tolerogenic effect of DMF might be related to the increased expression of inflammatory cytokines in the IEC, resulting in a reduced capacity by IEC to promote tolerogenic DC; and/or the increased frequency of *Il12*-expressing DC in the MLN results in reduced differentiation to Treg cells and, thereby, reduced frequency of Treg cells migrating back to the LP. In this context, it is interesting to note that IEC isolated from patients with the inflammatory bowel disease, Crohn’s disease, have a highly reduced ability to induce Treg-cell-promoting DC, as well as reduced *Aldh1a2* and *Tgfb1* expression as compared to healthy controls (77). Similarly, CD103+ DC isolated from colitic mice promote the generation of IFN-γ-producing CD4+ T cells rather than Foxp3+ Treg cells emphasizing the strong influence of the environment on CD103+ DC functionality (52).

In conclusion, we have demonstrated that fumarate signaling through HCAR2 mediated the activation of different pathways leading to different outcomes in different cell types, depending on the experimental and/or pathogenic environment. While DMF treatment of EAE-affected mice did not apparently alter gut morphology, it impacted upon the immunological profile, reverting some of the inflammatory parameters dysregulated in EAE, while enhancing others. Further studies in humans are necessary to understand whether or not the pleiotropic signaling *via* HCAR2 could be exploited to devise new lead compounds with reduced side effects.

## Data Availability Statement

The original contributions presented in the study are included in the article/**Supplementary Material**. Further inquiries can be directed to the corresponding author.

## Ethics Statement

The animal study was reviewed and approved by the Animal Ethics Committee of Ospedale Policlinico San Martino and by the Italian Ministry of Health (Approval Number: 398; authorization n. 679/2016-PR).

## Author Contributions

All authors had full access to all the data in the study and take responsibility for the integrity of the data and the accuracy of the data analysis. BP contributed to conception and design, collection and/or assembly of data, data analysis and interpretation, manuscript writing. AS contributed to collection and/or assembly of XPCT data. AC contributed to conception and design, data analysis and interpretation of XPCT analysis. AU revised the manuscript. NK contributed to conception and design, data analysis and interpretation, and manuscript writing and edition. All authors contributed to the article and approved the submitted version.

## Funding

This work was supported by a Basic Science Study grant from Biogen to A.U., by the FISR Project “Tecnopolo di nanotecnologia e fotonica per la medicina di precisione” (funded by MIUR/CNR, CUP B83B17000010001) and the TECNOMED project (funded by Regione Puglia, CUP B84I18000540002) to A. C., and by intramural funds from the University of Genoa.

## Conflict of Interest

AU has received consulting honoraria including as part of advisory board and/or speaker fees from Sanofi Genzyme, Roche, Biogen, Novartis, TEVA and Merck. This work was supported in part by a Basic Science Study grant from Biogen to AU.

The remaining authors declare that the research was conducted in the absence of any commercial or financial relationships that could be construed as a potential conflict of interest.

## Abbreviations

MMF, monomethyl fumarate; DMF, dimethyl fumarate; MS, multiple sclerosis; HCAR2, hydroxycarboxylic acid receptor 2; DC, dendritic cells; IEC, intestinal epithelial cells; EAE, experimental autoimmune encephalomyelitis; NF-kB, nuclear factor kB; DC, dendritic cells; AMPK, AMP-activated protein kinase; Sirt1, Sirtuin1; NA, nicotinic acid; COX-2, cyclooxygenase-2; BM, bone marrow; FBS, fetal bovine serum; sDC, splenic DC; LPS, lipopolysaccharide; GAPDH, glyceraldehyde 3-phosphate dehydrogenase; IFNg, interferon-g; PFA, paraformaldehyde; XPCT, X-ray phase contrast tomography; LP, lamina propria; MLN, mesenteric lymph node; FACS, fluorescence-activated cell sorting; CCR7, C-C chemokine receptor type 7; Cdh-1, Ecadherin; CCL19, chemokine (C–C motif) ligand 19; TGFb, transforming growth factor b; Aldh1a2, aldehyde dehydrogenase; DRF, diroximel fumarate.
