# Supplementary material for: Hydroxycarboxylic Acid Receptor 2, a Pleiotropically Linked Receptor for the Multiple Sclerosis Drug, Monomethyl Fumarate. Possible Implications for the Inflammatory Response
Source: Front Immunol. 2021 May 18;12:655212. doi: 10.3389/fimmu.2021.655212 (PMC8167049; doi:10.3389/fimmu.2021.655212)
Supplement: Supplementary file 1 [file DataSheet_1.pdf]

Supplementary Material

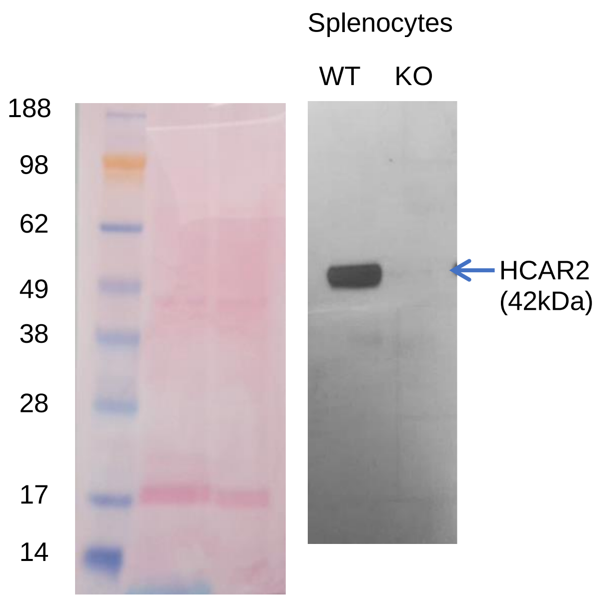

Supplementary Figure 1. HCAR2-KO mice do not express HCAR2 protein. Western blot analysis for HCAR2 in splenocytes isolated from WT and HCAR2-KO mice. One representative image of Western blot and Ponceau S staining of the membrane are shown

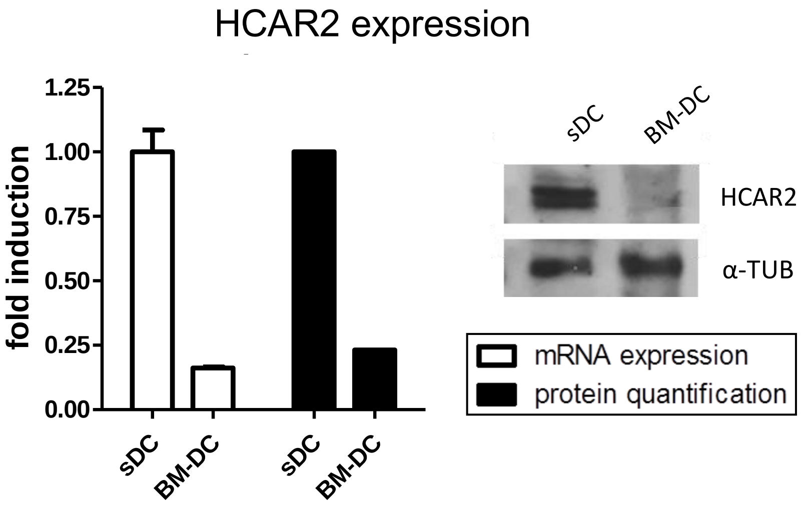

Supplementary Figure 2. HCAR2 is expressed at higher levels in sDC than in BM-DC. HCAR2 expression was assessed by RT-PCR (at mRNA level) and by Western blot (at protein level, using anti-HCAR2 and anti α-Tub antibodies) in sDC and BM-DC. Real time PCR data are presented as fold induction of gene expression in sDC over gene expression in BM-DC. Results are shown as mean ± SEM of two independent experiments. Quantification of Western blot by densitometric analysis of the bands is presented as the proportion of HCAR2 over α-Tub. One representative experiment is shown

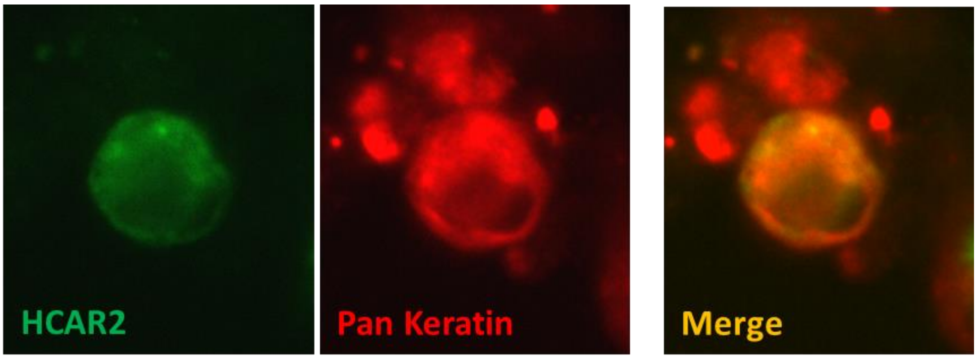

Supplementary Figure 3. IEC express HCAR2. Fluorescent microscopy images of in vitro-cultured IEC from naïve mice were performed using anti-HCAR2 (green) and anti-pan-keratin (red) antibodies. One representative experiment is shown
